# An intronic variant in *TBX4* in a single family with variable and severe pulmonary manifestations

**DOI:** 10.1038/s41525-023-00350-3

**Published:** 2023-03-06

**Authors:** Frances O. Flanagan, Alexander M. Holtz, Sara O. Vargas, Casie A. Genetti, Klaus Schmitz-Abe, Alicia Casey, John C. Kennedy, Benjamin A. Raby, Mary P. Mullen, Martha P. Fishman, Pankaj B. Agrawal

**Affiliations:** 1 Division of Pulmonary Medicine, Department of Pediatrics, Boston Children’s Hospital, and Harvard Medical School, Boston, MA USA; 2 Division of Genetics & Genomics, Department of Pediatrics, Boston Children’s Hospital, and Harvard Medical School, Boston, MA USA; 3 Department of Pathology, Boston Children’s Hospital, and Harvard Medical School, Boston, MA USA; 4 The Manton Center for Orphan Disease Research, Boston Children’s Hospital, Boston, USA; 5 Division of Newborn Medicine, Department of Pediatrics, Boston Children’s Hospital, and Harvard Medical School, Boston, MA USA; 6 Department of Cardiology, Boston Children’s Hospital, and Harvard Medical School, Boston, MA USA; 7 Division of Neonatology, Department of Pediatrics, University of Miami Miller School of Medicine, Miami, FL USA

**Keywords:** Disease genetics, Medical genetics

## Abstract

A male infant presented at term with neonatal respiratory failure and pulmonary hypertension. His respiratory symptoms improved initially, but he exhibited a biphasic clinical course, re-presenting at 15 months of age with tachypnea, interstitial lung disease, and progressive pulmonary hypertension. We identified an intronic *TBX4* gene variant in close proximity to the canonical donor splice site of exon 3 (hg 19; chr17:59543302; c.401 + 3 A > T), also carried by his father who had a typical *TBX4*-associated skeletal phenotype and mild pulmonary hypertension, and by his deceased sister who died shortly after birth of acinar dysplasia. Analysis of patient-derived cells demonstrated a significant reduction in *TBX4* expression resulting from this intronic variant. Our study illustrates the variable expressivity in cardiopulmonary phenotype conferred by *TBX4* mutation and the utility of genetic diagnostics in enabling accurate identification and classification of more subtly affected family members.

## Introduction

Pulmonary arterial hypertension (PAH) is a rare condition presenting in infants and children with a prevalence of 4.8–8.1 cases per million and despite advances in diagnosis and management, PAH continues to be associated with a high morbidity and mortality^1^. Unraveling the genetics of both sporadic and familial PAH will improve our understanding of the molecular basis of PAH and inform the development of novel therapeutics. Heterozygous variants in *BMPR2* were identified as the first monogenic cause of PAH and the number of genetic associations has rapidly increased over time. Whole exome and genome sequencing on large PAH cohorts has demonstrated an overall diagnostic yield of ~20% that may be as high as 40% in the pediatric population^2–6^

Heterozygous variants in the T-box factor 4 (*TBX4* gene) are the second leading genetic cause of PAH^2,3,7^. *TBX4* encodes for a transcription factor that is essential for limb development and multiple aspects of lung morphogenesis as revealed in animal models^8–11^. In 2004, heterozygous variants in *TBX4* were first associated with a spectrum of limb and skeletal anomalies referred to as small patella syndrome, also known as ischiocoxopodopatellar syndrome (OMIM 147891)^12^. The association of *TBX4* variation and PAH was later uncovered in 2013 and the phenotype has since expanded to include developmental lung abnormalities including acinar dysplasia, pulmonary veno-occlusive disease, and a spectrum of interstitial lung diseases^13–18^. A broad range of pathogenic variants has been observed in *TBX4*, including missense variants, nonsense/frameshift changes, and chromosome microdeletions. Intronic variants are also associated with ischiocoxopodopatellar syndrome as well as *TBX4*-related pulmonary hypertension^12,19^. A recent genotype-phenotype study demonstrated both loss-of-function and gain-of-function effects of *TBX4* variation and that those with haploinsufficiency present at earlier ages and with an increased incidence of interstitial lung disease than those with gain-of-function variants^17^. Herein, we report a novel *TBX4* intronic variant that causes reduced *TBX4* gene expression leading to variable and extreme phenotypes in a single family.

## Results

### Case report

The proband was a male born at term with respiratory failure. Soon thereafter, he developed bilateral pneumothoraces and was noted to have suprasystemic pulmonary hypertension with right-to-left flow across a large patent ductus arteriosus. He was initially treated with inhaled nitric oxide and later transitioned to oral sildenafil. By age 3 months, he was weaned off mechanical ventilation when an echocardiogram showed right ventricular pressure less than half of systemic pressure. He was discharged home, self-ventilating on room air with no requirement for pulmonary hypertension medications.

**Fig. 1:**
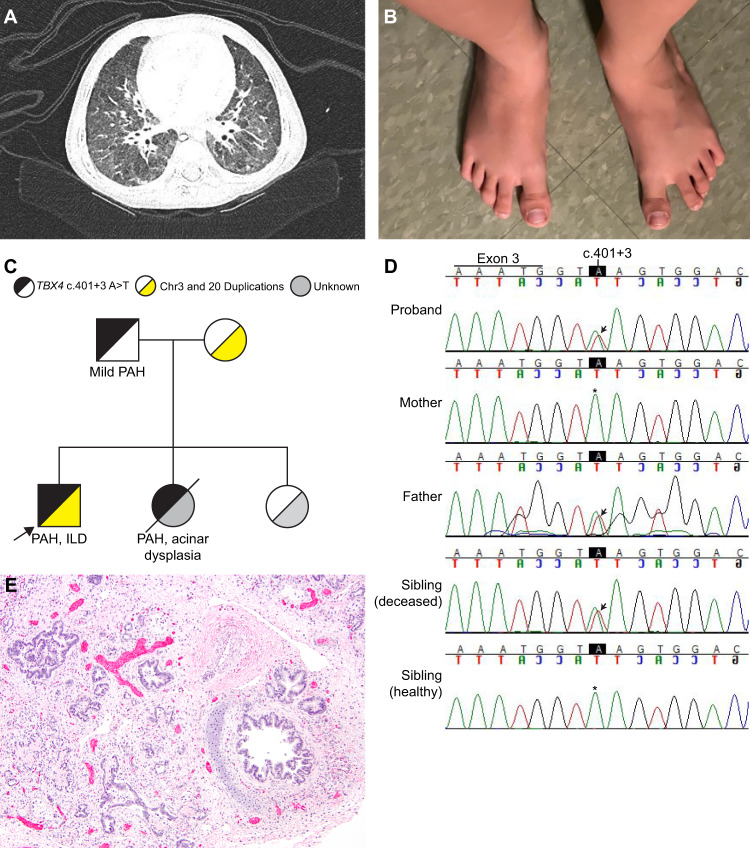
Clinical features of proband with *TBX4* intronic variant. **A** Chest CT at age 15 months showing perihilar peribronchial wall thickening with a paucity of peripheral lung markings. **B** Feet morphology showing widely spaced first and second toes bilaterally. **C** Family pedigree demonstrating inheritance of the *TBX4* c.401 + 3 A > T variant and the chromosome 3 and 20 duplications. Arrow denotes the proband. Abbreviations: PAH, pulmonary arterial hypertension; ILD, interstitial lung disease. **D** Sanger sequencing of *TBX4* in family. Arrows depict the c.401 + 3 A > T variant in the proband, father, and deceased sibling. The * denotes the typical sequence in the mother and healthy sister. **E** Postmortem lung showing nearly absent alveolar development in the proband’s sister, who died at 2 days of age (hematoxylin and eosin; original magnification, 40x).

At age 15 months the proband returned for follow-up and was noted to be intermittently tachypneic. Echocardiogram and cardiac catheterization showed pulmonary hypertension, and sildenafil was reinitiated. A chest CT showed a paucity of lung markings and perihilar peribronchial wall thickening (Fig. 1A). Over the subsequent 2 years, his lung disease progressed, with a 4 L oxygen requirement and moderate pulmonary hypertension treated with tadalafil and bosentan. Spirometry was consistent with restrictive lung disease, with reduced FVC at 0.8 L (0.8 L; 66% predicted), FEV1 of 0.78 L (0.78 L; 71% predicted), and high FEV1/FVC ratio (0.97). Neither lung volumes nor diffusion capacity could be assessed due to his young age. Throughout this time his physical examination was notable for retractions with exercise, lung fields clear to auscultation, and widely spaced first and second toes that is typically observed in *TBX4*-related ischiocoxopodopatellar syndrome with or without pulmonary arterial hypertension (Fig. 1B).

### Genetic testing results

Initial testing for genetic causes of respiratory disease and PAH (including sequencing for *FOXF1*, *SFTPB*, *SFTPC*, *ABCA3*) was negative. A chromosomal microarray (CMA) revealed two copy number variants (CNV); 3.2 Mb gain (3 copies) on chromosome 3p12.3-p12.2 (hg19; chr3:79216405–82396278), including two genes *ROBO1*, *GBE1* and a ~300 Kb gain (3 copies) on chromosome 20p13 (hg19; chr20:859816–1171343), both inherited from the mother and considered to be of unclear significance. The family was enrolled in the IRB-approved Gene Discovery Core protocol of the Manton Center for Orphan Disease Research for determination of an underlying genetic cause. Subsequently, whole-exome sequencing on a research platform revealed a novel intronic variant in *TBX4* in close proximity to the canonical donor splice site of exon 3 (hg 19; chr17:59543302; c.401 + 3 A > T) that was paternally inherited (Fig. 1C, D). It was not observed in the 141,456 subjects in the gnomAD database and is predicted to affect splicing by its position at the highly-conserved 3^rd^ base position of the donor splice site and Alamut visual version 2.11 (Interactive Biosoftware, Rouen, France) for bioinformatic splice prediction^20^. The mutation was confirmed clinically by a CLIA-certified laboratory. The whole-exome sequence also identified the CNV on chromosome 3 previously seen by CMA. The variant was of 6.9 Mb size (hg19; chr3:78,987,751-85,851,346) containing *CADM2*, *GBE1* and *ROBO1* (not full length, involves only exons 1–16 of 31 exons; *ROBO1* full length coordinates are chr3:78,646,388–79,817,059) genes and was also present in the mother (Fig. 1C).

### Family history and familial testing for TBX4 splice variant

Family history was notable for death of the proband’s sister on day 2 of life with an intracranial hemorrhage on ECMO, after presenting with profound hypoxemia, pneumothoraces and persistent pulmonary hypertension of the newborn; autopsy showed no substantial alveolar development, consistent with acinar dysplasia (Fig. 1E). The proband’s father (age 33 year at last visit) had foot morphology similar to the proband and reported asthma-like symptoms with full pulmonary function testing confirming moderate obstructive lung disease without any restriction or significant diffusion defect and only mild bronchial wall thickening on CT chest. He subsequently underwent a transthoracic echocardiogram that showed ventricular septal distortion during systole consistent with mild pulmonary hypertension. Sanger sequencing of the DNA extracted from the deceased sister’s postmortem lung tissue confirmed the same *TBX4* variant. Neither the proband’s mother nor his living sister carried the *TBX4* variant; both were healthy without skeletal or respiratory findings at last follow-up. The DNA obtained from the deceased sibling was of inadequate quality to evaluate for copy number change on chromosome 3 which was seen in proband and the mother.

### Reduced TBX4 expression resulting from novel splice variant

**Fig. 2:**
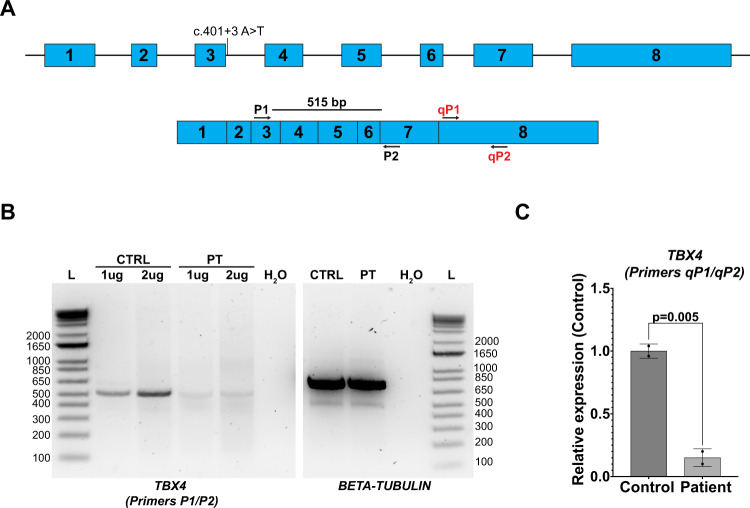
Reduced *TBX4* expression in proband-derived lymphoblastoid cell lines. **A** Schematic of *TBX4* genomic structure (top) and spliced cDNA (bottom). Primer pairs used in B and C are depicted. **B** PCR analysis of *TBX4* transcript using primers P1 and P2 on cDNA derived from 1ug or 2ug of RNA isolated from control (CTRL) or proband-derived (PT) lymphoblastoid cell lines (left panel). PCR analysis of *β-tubulin* transcript was used as a control (right panel). H_2_O denotes water control and L denotes the 1 Kb ladder. **C** qPCR analysis of *TBX4* expression using primers qP1 and qP2 on control and proband-derived lymphoblastoid cell lines. Data is normalized to *GAPDH* expression and is expressed relative to control sample expression. Two biologic replicates included per experiment that were repeated *N* = 2. *P*-value determined by Student’s 2-tailed *t*-test. Error bars reflect standard deviation.

As lung tissue from the proband was not available for analysis of *TBX4* alternative splicing, we generated lymphoblastoid cells lines from peripheral blood mononuclear cells (PBMC) isolated from the proband and a healthy control for analysis of *TBX4* alternative splicing resulting from the c.401 + 3 A > T variant. This approach was based on prior studies that were able to detect *TBX4* expression from EBV-transformed lymphoblastoid lines to reveal pathogenic alternative splicing in a family with ischiocoxopodopatellar syndrome^12,19^. The exon-spanning primers used for these studies are depicted in Fig. 2A with respect to the *TBX4* cDNA. While *TBX4* expression was not detected from primary PBMCs (data not shown), the expected 515 bp band was amplified by PCR from cDNA derived from the lymphoblastoid lines and sequencing confirmed the identity of this product as the correct *TBX4* sequence (Fig. 2B and data not shown). Smaller or larger molecular weight bands to implicate exon skipping or intron inclusion, respectively, were not observed in the proband’s sample by this method; however, *TBX4* transcript levels were reduced in the proband compared to control lymphoblastoid lines (Fig. 2B). To confirm this reduced expression, qPCR was performed on control- and proband-derived lymphoblastoid lines using primers to amplify exon 8. Of note, a prior study describes another intronic c.282-1 G > A variant that produced two alternatively spliced transcripts by RNA sequencing at low frequency including c.282delG and c.282_284delGAG identified in 0.7% and 3.3% of total reads, respectively^19^. This analysis confirmed reduced expression of *TBX4* in proband-derived lymphoblastoid cells compared to control cells (Fig. 2C), demonstrating a disruption of *TBX4* expression resulting from this variant.

## Discussion

We describe a proband and two of his first-degree relatives to have a splice-site point mutation in *TBX4*, the second most frequently implicated gene, after bone morphogenetic protein receptor-2 (*BMPR2*) in PAH^7^. Our family carried a novel splice site variant—a single nucleotide substitution at the third base position of donor splice site of intron 3. Although +3 position splice site variants represent <5% of pathogenic donor mutations, several lines of evidence support this c.401 + 3 A > T variant as pathogenic: (i) the segregation of this variant with *TBX4*-compatible phenotypes in three family members; (ii) the variant was not observed among 280,000 chromosomes in the gnomAD database; (iii) c.401 + 3 is highly evolutionarily conserved, with adenine observed in >90% of mammalian and reptilian species (and thymidine never observed); and (iv) we observed a significant reduction in *TBX4* expression in our proband-derived lymphoblastoid cells.

A striking feature exhibited by this variant is its variable expressivity. In addition to the characteristic skeletal feature of widely spaced first and second toes, our proband displayed the clinical phenotype associated with *TBX4*-induced pulmonary arterial hypertension—a biphasic course of neonatal respiratory failure and PPHN with apparent resolution, followed by chronic PAH later in infancy^6^. Our proband also has a lung phenotype consistent with a TBX-4 associated abnormality in lung development, supported by chest imaging and symptoms in the setting of known acinar dysplasia in deceased sister. In contrast, other family members harboring the mutation manifest two extremes of pulmonary phenotype severity: the proband’s sister manifested a diffuse developmental lung abnormality (acinar dysplasia) and severe pulmonary arterial hypertension and died within 48 h of birth, while the father had skeletal findings similar to his son and evidence of mild pulmonary hypertension on echocardiogram. Pathologic studies to date indicate that *TBX4* mutation is manifested in the lung primarily as a diffuse alveolar growth abnormality ranging from mildly diminished alveolarization (alveolar simplification) to an acinar-dysplasia-like severe paucity of alveolar development, all accompanied by varying degrees of pulmonary arterial hypertensive remodeling likely due to a diminished pulmonary capillary bed^13–18^. The family described here illustrates the potential for single *TBX4* genotype to result in a wide spectrum of phenotypic expression even within the two generations of a single family. These observations show that the *TBX4* genotype is not always predictive of disease severity and that it is important to consider other genetic or epigenetic factors. Hence it is imperative to obtain a thorough family history (and, if possible, examine at-risk relatives) to identify more subtle presentations^21^.

While molecular analysis of patient-derived EBV lymphoblastoid lines did not identify splicing alterations as a result of the *TBX4* variant, qPCR demonstrated a reduction in overall *TBX4* expression in patient-derived lines. A recent study identified the c.282-1 G > A intronic *TBX4* variant in a 5-month-old girl that was inherited from an unaffected father with somatic mosaicism for this variant. Interestingly, this variant similarly produced reduced *TBX4* expression and RNA sequencing captured two alternatively spliced transcripts at low frequencies of 0.7% and 3.3% likely due to nonsense mediated decay^19^. This suggests that the c.401 + 3 A > T identified in this study may also produce alternatively spliced transcripts that were not detectable by RT-PCR due to nonsense mediated decay. Importantly, the magnitude of this reduction in *TBX4* expression is greater than anticipated for a heterozygous variant. A recent study identified a positive feedback loop wherein TBX4 protein binds upstream to the *TBX4* promoter to reinforce its expression^22^. This autoregulation may account for the observed differences in *TBX4* expression resulting from the c.401 + 3 A > T variant and it will be interesting to analyze *TBX4* expression in other families with loss-of-function variants.

We note that the proband’s large duplication on chromosome 3 carried from the mother and containing genes including *ROBO1* may potentially play a role in the severe phenotype of the proband. The gene product ROBO1 is a known regulator of endothelial cell function and may affect lung vascular development in conjunction with reduced *TBX4* expression^23–25^. There exists no defined anatomic correlate to mutations in *ROBO1* and therefore we are unable to evaluate whether the *ROBO1* duplication may have contributed to the phenotype.

In summary, we describe a family with two siblings affected by severe pulmonary hypertension and diffuse lung disease who carry a *TBX4* variant from the father. Our findings strongly support that the variant is pathogenic yet expressed with a wide range of severity.

## Methods

### Patient recruitment and genetic testing

The methods were performed in accordance with relevant guidelines and regulations and were approved by the Institutional Review Board at Boston Children’s Hospital. All participants gave written informed consent to participate in the research protocols. The chromosome microarray was performed as a clinical test at Claritas Genetics (Cambridge, MA). Whole-exome sequencing was performed on the DNA extracted from the trio at Broad Institute and data analyzed using the SEQR pipeline^26^. Variant confirmation was performed by GeneDx (Gaithersburg, MD).

### EBV-transformed lymphoblastoid cell line generation

PBMC were isolated from whole blood using Ficoll-Paque™ and SepMate™ tubes (Stem Cell Technologies, Vancouver, CA). Human B-lymphocytes were immortalized by transformation of 5 × 10^6^ PBMC with supernatant from B95.8 (ATCC) in the presence of 1 mg/mL of cyclosporine A (Sandimmune®, Novartis). EBV-LCLs of the patient and the control were cultured in advanced RPMI-1640 medium (ThermoFisher) supplemented with 10% fetal bovine serum (FBS, ThermoFisher), 2 mM GlutaMAX™ (ThermoFisher), Gibco™ Non-Essential Amino Acids, and 10 mM Gibco™ HEPES at 37 °C in a 5% CO2 atmosphere. Cells were maintained at a concentration between 5 × 10^5^–1 × 10^6^ cells/mL and expanded as needed.

### RNA extraction, cDNA synthesis, and PCR amplification

RNA was extracted using the RNeasy Plus Mini Kit (Qiagen) and genomic DNA was eliminated via gDNA eliminator column (Qiagen). cDNA was synthesized using the High Capacity cDNA Reverse Transcription Kit (Applied Biosystems). PCR products were amplified using the Expand High Fidelity PCR System (Roche). The following primers were used to amplify *TBX4* and β-tubulin.

*TBX4* Forward (P1): catgaaccccaagaccaagt; Reverse (P2): tctgcctcatgatgcttttg

*ß-Tubulin* Forward: tctgttcgctcaggtccttt; Reverse: aagcatctgctcatcgacct

### qPCR analysis of TBX4 expression

qPCR reactions were performed using the PowerUp SYBR Green Master Mix (ThermoFisher) and run on the QuantStudio 7 Flex RT-PCR System (ThermoFisher). Relative expression was normalized to GAPDH. The following primers were used:

*TBX4:* Forward (qP1): gcaagcgatcctatctggaa; Reverse (qP2): tgtacggcgacactgaagtc

*GAPDH:* Forward: gagtcaacggatttggtcgt; Reverse: tggaagatggtgatgggatt

## Data Availability

The datasets generated during and/or analyzed during the current study are available from the corresponding author on reasonable request. The variant information has been submitted to ClinVar (Accession: VCV001188835.1). Exome sequencing data cannot be shared due to privacy concerns, but can be requested from the corresponding author from a qualified researcher. Access to the exome sequencing data will require an IRB-approved collaboration and Data Usage Agreement.
